# Rumen Synergistota: new insights into their role in mimosine and fluoroacetate toxicity of ruminant livestock

**DOI:** 10.1128/aem.00380-25

**Published:** 2025-07-17

**Authors:** Christopher S. McSweeney, Michael Halliday, Roderick I. Mackie

**Affiliations:** 1 CSIRO Agriculture and Food, Queensland Biosciences Precinct, St Lucia, Brisbane, Queensland, Australia; 2 Zillmere, Brisbane, Queensland, Australia; 3 Department of Animal Science, and Carl R. Woese Institute for Genomic Biology, University of Illinois 166978, Urbana, Illinois, USA; University of Illinois Urbana-Champaign, Urbana, Illinois, USA

**Keywords:** Synergistota, *Synergistes jonesii*, *Cloacibacillus*, *Pyramidobacter*, mimosine, fluoroacetate, detoxification, rumien, dehalogenation

## Abstract

This paper examines several rumen bacteria in the Synergistota phylum, specifically focusing on their potential to detoxify harmful compounds found in plants grazed by ruminants. Synergistota bacteria ferment amino acids for energy, while rumen *Synergistes jonesii* from which the phylum was named, can also metabolize toxins found in the forage plant *Leucaena leucocephala* (leucaena). Specifically, *S. jonesii* is able to detoxify mimosine, a non-protein amino acid in leucaena, by converting it into less harmful metabolites. Historically, *S. jonesii* was introduced to ruminants in Australia to mitigate leucaena toxicity based on the notion that the bacterium was absent on this continent. Recent studies indicate geographic variations in *S. jonesii’s* presence and effectiveness, suggesting that strain variability may impact its detoxification efficacy. PCR-based assays have improved the detection of *S. jonesii*, revealing its widespread distribution in Australia and globally, but often low abundance in ruminant microbiomes. Additionally, other rumen Synergistota species (*Cloacibacillus porcorum* and *Pyramidobacter piscolens*) have recently been isolated and identified as agents for metabolizing fluoroacetate, another toxin present in Australian flora. These bacteria degrade fluoroacetate through a novel molecular mechanism of reductive dehalogenation, thus producing fluoride ions and acetate as byproducts. This mechanism has been detected in soil and contaminated groundwater but not the rumen. These findings underscore the ecological importance of Synergistota bacteria in reducing plant toxicity in ruminants. Ongoing research is recommended to isolate new strains, optimize rumen populations of these bacteria, and further understand the molecular pathways involved in toxin degradation to enhance detoxification capabilities in ruminant populations.

## INTRODUCTION

Foregut fermentation has evolved independently in four different classes of vertebrate animals, such as filter feeding whales, some marsupials and rodents, folivorous monkeys and hippos, and also in a bird, the hoatzin (1, 2). However, the most recent and most specialized evolutionary adaptation to the mammalian mutualistic gut ecosystem is the ruminant animal (1, 3). Ruminants are characterized by large pre-gastric fermentation chambers that store the ingesta and slow its rate of passage, allowing a dense and diverse rumen microbial community to digest feedstuffs that are indigestible to the host animal. This process is greatly assisted by the process of rumination, in which a bolus of ingested feed is regurgitated, mixed with saliva, and re-masticated before being swallowed. This enables a reduction in particle size, termed comminution, resulting in exposure of a large surface area of feed particles for microbial attack. Of importance to this review is the ability and capacity of rumen microorganisms, particularly anaerobic bacteria, to adapt to and metabolize toxic plant materials to harmless compounds (2, 4).

**Fig 1:**
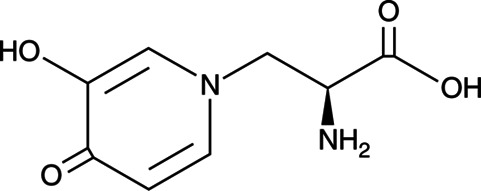
Mimosine {*β*-[*N*-(3-hydroxy-4-oxypyridyl)]-*α*-aminopropionic acid}.

**Fig 2:**
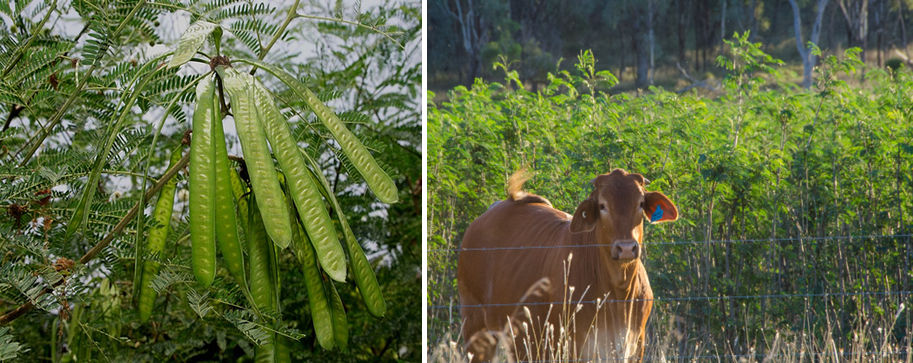
The tropical leguminous shrub *Leucaena leucocephala.*

Ruminants are best known for their ability to digest fibrous, low-protein feeds that are indigestible by most non-ruminant animals. In this mutually beneficial relationship between the ruminant animal and foregut (pre-gastric) fermentation by anaerobic bacteria, methanogenic archaea, ciliate protozoa, and anaerobic fungi, an eco-physiological advantage is provided to the host animal through the supply of nutrients (energy from volatile fatty acids, protein from microbial cells, and B-vitamins synthesized by bacteria). However, possibly one of the most important reasons for the evolution of foregut fermentation is the detoxification of plant toxins (phytotoxins) and fungal toxins (mycotoxins) that occur in the feed. Thus, it has been suggested that ruminants have greater flexibility and chance of survival when diet choice is limited, due to pre-gastric microbial detoxification of phytotoxins and mycotoxins that are poisonous to their non-ruminant competitors. Phytotoxins occur in a wide variety of feeds, and in many instances, the ruminant forestomach provides a protective function as the ruminal microbiota effectively degrade a wide variety of toxic compounds. In this review, we focus on two compounds that occur in plants that are consumed by ruminants in different parts of the world (5). The first is a non-protein amino acid, named mimosine (Fig. 1) chemically similar to tyrosine, that occurs in a tropical leguminous shrub *Leucaena leucocephala* (Fig. 2) that is widely used as a nitrogen supplement, in the form of a protein bank, but is toxic to ruminants in some parts of the world (6). Mimosine is usually highest in young and actively growing parts of the plant and can constitute more than 2%–20% of the dry matter depending on the stage of growth (7).

The second toxin is a fluorinated acid (fluoroacetate), where detoxification involves reductive dehalogenation. Plants that contain monofluoroacetate (FA; CH2_2_F-COO--^--^) grow worldwide and cause losses in productivity and acute death in ruminant livestock. The southern continents of tropical and sub-tropical Australia, Africa, and South America are the main geographical regions where these plants grow and cause losses in ruminant livestock grazing under extensive/range production systems (8). In Australia, most plants containing fluoroacetate belong to the genera *Gastrolobium* and *Acacia*. The main plants that cause fluoroacetate toxicity in Brazil and South Africa are *Palicourea marcgravii* and *Amorimia rigida* (in Brazil) and *Dichapetalum cymosum* (in South Africa). Fluoroacetate is found in the leaves and seeds of these plants, and high concentrations ( >2,000 mg/kg) usually occur in young regrowth (8).

**Fig 3:**
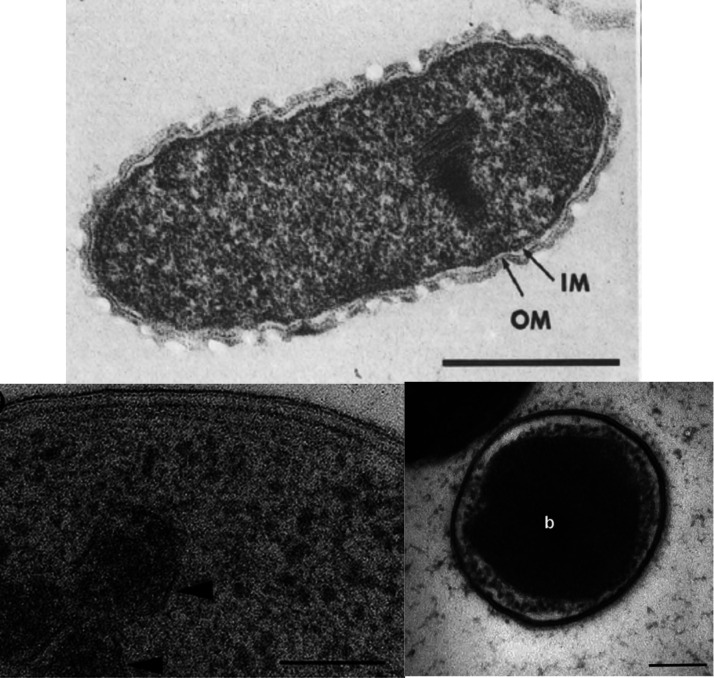
Electron micrographs. (Top) *Synergistes jonesii*, with arrows designating outer membrane (OM) and inner membrane (IM) (bar, 500 nm) (reprinted from reference 10, published under the terms of the Creative Commons License, https://creativecommons.org/licenses/by/4.0/). (Bottom left) *Cloacibacillus pocorum* strain MFA1, showing gram-negative cell wall and electron-dense granules with surrounding membrane (arrowheads) (bar, 100 nm) (reprinted from reference 11 with permission of the publisher). (Bottom right) *Pyramidobacter piscolens* C12-8, showing the unusual single membrane cell envelope and the central electron-dense body (b) (bar, 200 nm) (reprinted from reference 12 with permission of the publisher).

Interestingly, although these toxins belong to widely different chemical classes, the anaerobic bacteria that degrade and modify these compounds are found in the same bacterial phylum, Synergistota. This phylum of bacteria shares phenotypic characteristics including a gram-negative type of cell wall, anaerobic lifestyle, and rod/vibrioid cell shape (Fig. 3). Most Synergistota are asaccharolytic, but all share the ability to ferment amino acids (9).

This mini review explores the role of rumen bacteria within the Synergistota phylum, particularly their ability to detoxify harmful compounds such as mimosine and fluoroacetate in plants consumed by ruminants. Also presented are historical events that led to the discovery of rumen Synergistota species and some controversies regarding their role in reducing plant toxicity in ruminants, including overall low abundance in the rumen, limitations of analytical procedures for detecting dihydroxypyridone (DHP), strain-to-strain variation, and possible loss of activity in conditions where substrates are absent or at low concentrations.

## SYNERGISTOTA BACTERIA

**Fig 4:**
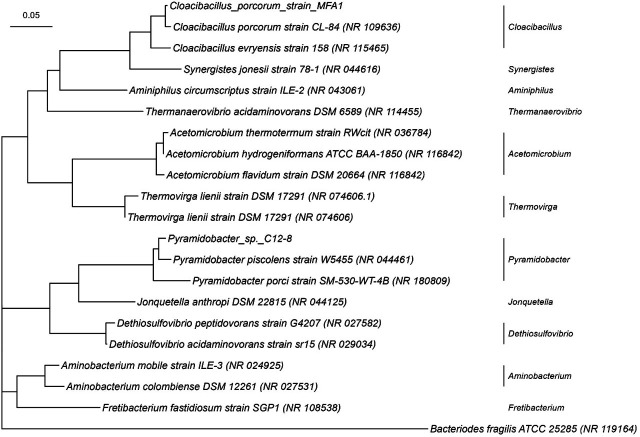
Phylogenetic tree of Synergistota based on near full-length 16S rRNA gene sequences constructed using the maximum likelihood method and Tamura-Nei model (18). *Bacteroides fragilis* is included as an outgroup. This analysis involved 21 nucleotide sequences from GenBank (accession number), including two isolates from CSIRO (strains MFA1 [19] and C12-8 [12]). Evolutionary analyses were conducted in MEGA X (20), and the Newick tree format was generated. The tree is drawn to scale (left top), with branch lengths measured in the number of substitutions per site by “*ggtree*” in R package (21).

The Synergistota phylum has an interesting taxonomic history. The phylum was named after the first species isolated, *Synergistes jonesii*, which originated from the rumen of a goat (10). Within the phylum, there is a single class Synergistia and one order (*Synergistales*) containing 22 genera as representatives of eight families (*Acetomicrobiaceae*, *Aminiphilaceae*, *Aminobacteriaceae*, *Dethiosulfovibrionaceae*, *Synergistaceae*, *Thermovirgaceae*, and two yet to be named families) (13). A phylogenetic tree of Synergistota genera based on near full-length 16S rRNA gene sequences is shown in Fig. 4. Synergistota are found in a wide range of anaerobic habitats, including animal and insect gastrointestinal tracts, wastewater treatment systems, soils, and oil wells, and they have also been implicated in opportunistic pathogenic diseases. They are usually present as a minor population (<1%) in these ecosystems (14). Despite this habitat diversity, isolates bear a striking physiological resemblance to one another in that they all ferment amino acids rather than carbohydrates and have the highest average proportion of amino acid transport and metabolism genes in any bacterial phylum to date (15–17).

One possible explanation for this apparent physiological uniformity is that members of the Synergistota have diversified by exploiting the same type of metabolic niche (generalized amino acid fermentation) in different environments. The few cultured isolates from the phylum also display differing abilities to use secondary plant compounds (hydroxypyridone, FA) as electron acceptors to facilitate fermentation of amino acids, which are their primary carbon source (10, 11). An uncultured *Synergistes* sp. L4M2 (clone 196.B09) from an enrichment of ovine rumen fluid was also identified as a bacterium that may be able to detoxify hepatotoxins from the pyrrolizidine alkaloid family (22).

In the rumen, two genera (*Pyramidobacter* and *Synergistes*) belonging to the *Synergistaceae* family*, and Cloacibacillus* and a clade named TG5, both belonging to the family Dethiosulfovibrionaceae, have been identified in multiple ruminant species including sheep, goats, cattle, and deer (12, 23). In the global rumen census of ruminants, only *Pyramidobacter* and clade TG5 were identified as minor rumen populations (<1%), and the *Synergistaceae* were not reported, presumably due to the depth of pyrosequencing of samples (23). Synergistota bacteria also appear to represent a small proportion of the bacteria that colonize the rumen epithelium (24). We have observed in cattle and goats from Australia and Thailand, respectively, that *Pyramidobacter* and the TG5 clade are consistently the dominant Synergistota, while members of the *Synergistaceae* (*Synergistes* sp. and *Cloacibacillus* sp.) are minor populations. In rumen batch cultures, the abundance of the Synergistota phylum increased substantially from 0.1% to 4.6% in response to ginkgo plant extract (containing alkyl phenols), and *Pyramidobacter* was the dominant member (25).

## LEUCAENA TOXICITY IN RUMINANTS

### Early history

**Fig 5:**
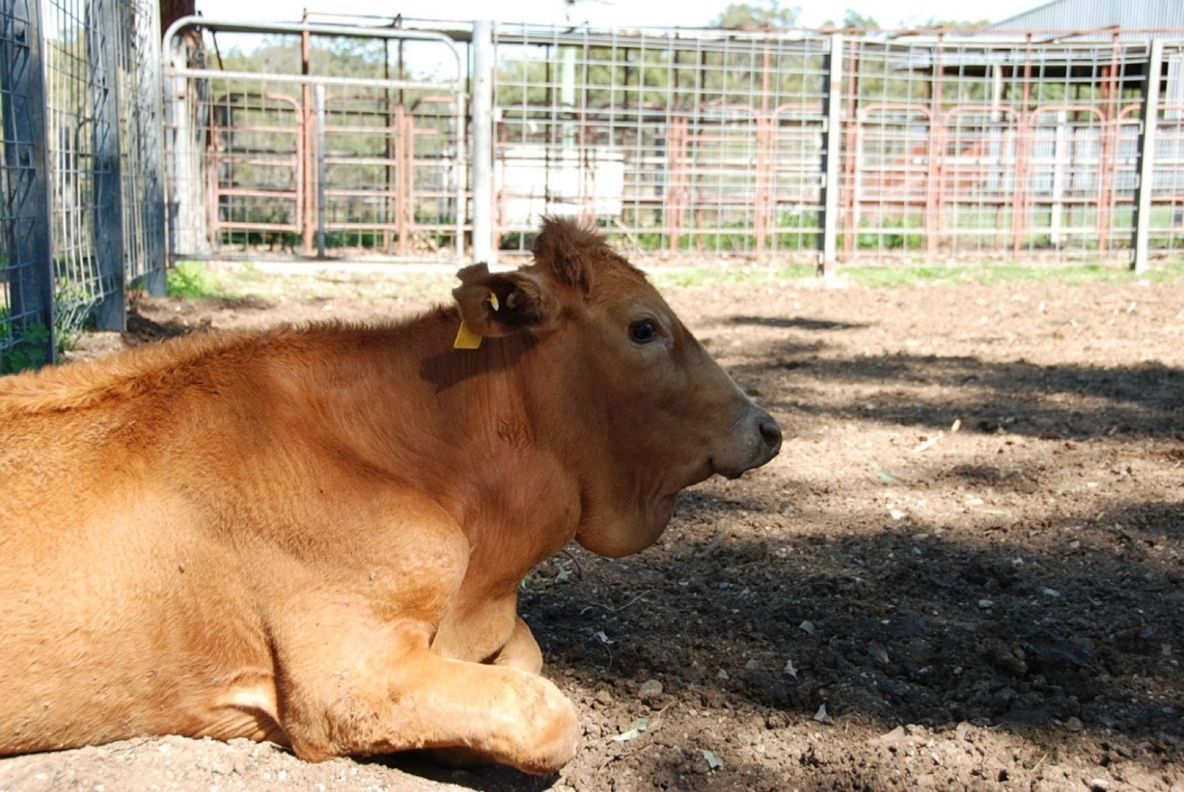
Goiter in a leucaena-intoxicated bovine.

Isolation of the first bacterium (*S. jonesii*) from the phylum occurred during the early 1990s and arose in response to the problem of toxicity observed more than 10 years earlier in Australian ruminants consuming the highly valued forage-tree legume *Leucaena leucocephala* (leucaena) (10). The plant is native to Central America but now grows globally in tropical regions where ruminants graze and in smallholder cut-and-carry systems. It is invaluable as a protein supplement due to the low-nitrogen content of native forage plants in these regions. However, it contains the non-protein toxic amino acid mimosine {β-[*N*-(3-hydroxy-4-oxopyridyl)]-α-aminopropionic acid} (26). Mimosine has acute anti-mitotic activity that affects rapidly dividing cells (27, 28), but the acute toxicity is usually managed by introducing animals to the plant gradually. This enables the rumen microorganisms in general to adapt and rapidly degrade the amino acid to the slower-acting toxic metabolite 3,4-DHP (3-hydroxy-4[1*H*]-pyridone), which is goitrogenic (26, 29). Hegarty and co-workers (30, 31) were the first to identify 3,4-DHP as the primary metabolite of mimosine metabolism in the rumen. Research into leucaena toxicity gained prominence in Australia during the 1970s after the plant was introduced into cattle grazing systems in northern Queensland, and symptoms of toxicity—including alopecia, loss of appetite, enlarged thyroids (Fig. 5), and reduced thyroxine—were reported (28, 31–34). However, signs of toxicity were not evident in many other countries where leucaena was fed to ruminants, leading to the conclusion that some ruminants were not susceptible (33). Consistent with this view, Raymond Jones from the Australian Commonwealth Scientific and Industrial Research Organization (CSIRO) observed that goats grazing leucaena in Hawaii were not intoxicated and postulated that further rumen metabolism of the toxins protected these animals (35). Subsequently, R. J. Jones and J. B. Lowry (36) reported that Australian goats imported into Indonesia, where chronic leucaena toxicity was not evident, were able to detoxify the 3,4-DHP after rumen infusion from an Indonesian goat. Detoxification was based on the observation that urinary excretion of DHP declined markedly following infusion of the rumen fluid. Furthermore, R. J. Jones and R. G. Megarrity (37) demonstrated that an *in vitro* mixed culture of rumen microorganisms from a Hawaiian goat that could metabolize both 3,4- and 2,3-DHP (3-hydroxy-2[1*H*-pyridone) protects cattle in Australia from leucaena toxicity when inoculated into their rumen. The presence of the 2,3-DHP isomer as a metabolite in the urine of leucaena-fed ruminants was first reported by C. W. Ford et al. (38) but was regarded as only sporadically present.

### Identification of DHP-degrading bacteria

**Fig 6:**
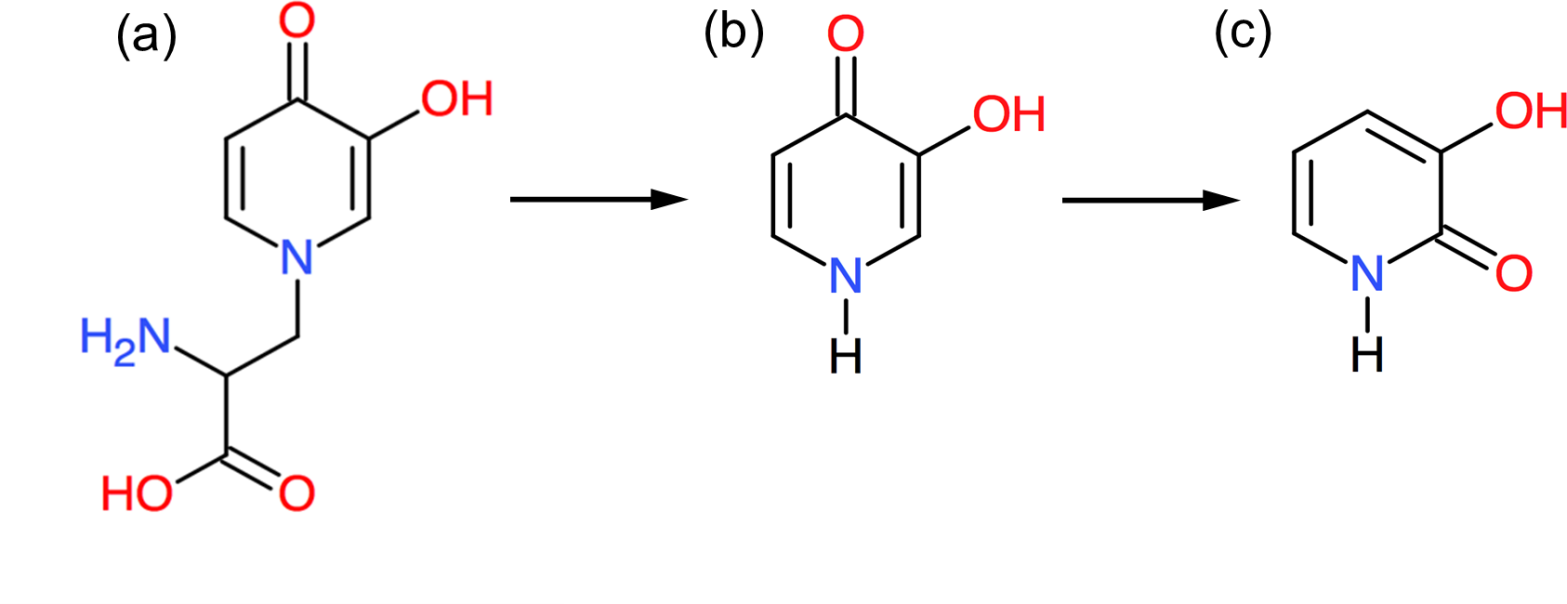
Ruminal degradation pathways of (**a**) mimosine; (**b**) 3,4-DHP; and (**c**) 2,3-DHP (adapted from reference 40, published under the terms of the Creative Commons License, https://creativecommons.org/licenses/by/4.0/).

These findings provided the impetus for Milt Allison from the United States Department of Agriculture to isolate four DHP-degrading bacteria from the mixed rumen culture used in Australian cattle that originated from the Hawaiian goat (10). The four strains of bacteria were very similar genetically, obligately anaerobic, gram-negative, rod-shaped, and could isomerize 3,4-DHP to 2,3-DHP prior to cleaving the pyridine ring to non-toxic metabolites such as short-chain fatty acids (Fig. 6). One of the bacteria, strain 78–1 was characterized in detail and assigned to a new genus and named *S. jonesii* as the type strain (ATCC 49833). Hence, the paradigm emerged that bacteria with the ability to degrade 3,4-DHP via isomerization to 2,3-DHP were not ubiquitous, and this rumen metabolic capacity was geographically limited to ruminant populations in different regions of the world. It was proclaimed that ruminants in Australia lacked native populations of *S. jonesii*, which could be rectified by colonizing the rumen with *S. jonesii* introduced from the Hawaiian goat. However, it is not clear from publications whether urinary 2,3 DHP was present in Australian cattle prior to the release of *S. jonesii* in the 1980s, which would have indicated more extensive rumen metabolism of 3,4-DHP than previously thought. Consequently, a commercial inoculum was developed in Australia that involved the transfer of fresh rumen fluid from *S. jonesii* colonized cattle to naive animals. Later, a rumen inoculum taken from a steer exposed to animals that had received the rumen culture from the Hawaiian goat was maintained in several continuous cultures for a period of 9 days with inoculum being harvested over the final 3 days and used as an “oral cattle drench” in northern Australia, with few subsequent cases of toxicity but some producers report poor productivity responses (39). Nevertheless, this has led to endless speculation that other detoxifications and important metabolic activities, such as specific elements of carbohydrate and nitrogen digestion, may be absent in geographically isolated ruminant populations, which could be rectified by introducing microorganisms from ruminants with the desired rumen phenotype. However, to the best of our knowledge, there are no published reports of this being achieved or additional evidence for the limited distribution of unique metabolism in ruminants.

### Recent leucaena toxicity research and insights

For many years, it was generally regarded that the problem of leucaena toxicity in Australia had been solved with the widespread use of the commercial inoculum containing *S. jonesii*. However, early in the new millennium, reports emerged of some cattle deaths and symptoms of DHP toxicity in cattle browsing lush leucaena in Australia. This led to a survey of the urinary DHP levels in more than 40 cattle herds previously inoculated with *S. jonesii* and grazing leucaena (41). Nearly 50% of the herds had urinary concentrations of DHP >100 µg/mL, which was indicative of subclinical toxicity, and high levels of both 3,4- and 2,3-DHP were often detected. Similarly high levels of urinary DHP (3,4- and 2,3-DHP) have been observed in village goats, cattle, and buffalo herds in India, eastern Indonesia, and Thailand without signs of toxicity (42–44).

In response to these findings, new PCR-based assays were developed and refined over several years using both 16S rDNA/rRNA as template for the detection of *S. jone*sii (45–47). Initially, to improve the sensitivity and specificity of detection, Graham et al. (45) used a nested PCR approach to monitor the presence of *S. jonesii* in cattle from several northern Australian properties. Less than 10% of the cattle tested were positive for *S. jonesii*, even though DHP degradation was evident both before and after inoculation with the bacterium. Using a similar assay for a survey of several ruminant species resident in different countries, it was concluded that *S. jonesii* appears to be indigenous to all types of ruminants and geographical regions (46). Later, using even more sensitive RNA-based RT-PCR assays for a survey of ruminants from different countries supported the observation that *S. jonesii* is ubiquitous rather than isolated geographically (47). These surveys also confirmed that the bacterium is usually present as a minor population of about ≤10^6^ cells/mL of rumen fluid. *S. jonesii* has also been detected in several non-ruminant herbivores (6).

It is generally accepted that the ability to degrade DHP is dependent upon the presence of *S. jonesii* in the rumen, even though it has been reported that other bacteria (*Clostridium* spp., *Lactobacillus* spp., *Streptococcus* spp., and *Klebsiella* spp.) can degrade DHP (48–52). However, the ability of any of these isolates to detoxify DHP has not been verified independently, nor do the isolates still appear to be in collections. Nevertheless, it is feasible that bacteria other than *S. jonesii* exist that can metabolize either isomer of DHP or both. In this regard, M. J. Allison et al. (53) observed that rumen fluid from some cattle in Florida, USA, that did not have exposure to leucaena degraded 2,3-DHP but not the 3,4 isomer, while others degraded neither. These cattle had been introduced to the Sub-tropical Research Station in Florida from the US Virgin Islands. Cattle in tropical regions of the USA (US Virgin Islands) and Haiti where leucaena was growing were more likely to degrade both DHP isomers (53).

It is also possible that the ability of *S. jonesii* to degrade DHP may vary between strains of the species. While conducting surveys of *S. jonesii* internationally, it was observed that there was genetic variation at the species level based on characteristic single-point polymorphisms in specific regions of the 16S rRNA gene (46, 47). It is possible that the DHP-degrading ability of different strains may vary, particularly if some *S. jonesii* bacteria are present in ruminants where leucaena and DHP are absent from the environment. Furthermore, it has been shown that the ability of *S. jonesii* to degrade and thus detoxify 2,3-DHP can be lost permanently or temporarily if cultured in the absence of the toxin (54). The rate of isomerization of 3,4-DHP to 2,3-DHP and cleavage of the pyridine ring are also regulated by the concentration of pyridinediols (55).

Therefore, it would not be surprising if there are differences between animals in DHP degrading ability depending on variations in toxin intake due to season and growth conditions, the geographical region, and whether leucaena is naturalized in the environment or only recently introduced. Halliday and co-workers (56) have shown recently that this may be the case when they noted that following the dosage of Australian cattle with the commercial enrichment inoculum of *S. jonesii*, the extent of 2,3-DHP degradation appeared to increase, and total DHP excretion decreased further, indicating that the inoculum may have been more effective in degrading 2,3-DHP than the indigenous strains already present and degrading both isomers. One might conclude that, in certain circumstances, inoculation with *S. jonesii* strains that have been maintained in culture in the presence of both DHP isomers could augment the detoxifying potential of indigenous *S. jonesii*, especially if the animals had no prior exposure to leucaena. However, we should remain open to the notion that other species of DHP-degrading bacteria may exist in the rumen that contribute to these differences in the metabolism of the two DHP isomers. Another important aspect accounting for differences in susceptibility to toxins and variation in research outcomes may relate to the autolysis of mimosine during ingestion. Mimosine partially undergoes autolysis to 3,4-DHP, pyruvate, and ammonia by leaf enzymes during ingestion and mastication, with half the mimosine in macerated leaves being degraded in 4 minutes (30, 57). Thus, considerable conversion of mimosine to DHP occurs before the material even arrives in the rumen.

### Excretion of DHP  

Until recently, little attention was paid to the excretion of DHP in urine because the early reports indicated that after inoculation with *S. jonesii*, DHP excretion declined rapidly, and 2,3-DHP was a minor product or transient (38, 58). These observations differ from more recent studies of small and large ruminants browsing leucaena in Australia and other countries, which show that significant amounts of both DHP isomers are excreted in urine (6, 41, 45). Early studies with the type strain of *S. jonesii* (strain 78–1) in a mixed-rumen population chemostat suggested that the rate of degradation of DHP that occurred and low abundance of this organism (59) cannot be solely responsible for protecting animals that are consuming 100% leucaena, such as in cut and carry systems in tropical countries. These observations led Dr. Max Shelton from the University of Queensland and others to focus on the contribution of hepatic conjugation of DHP isomers and their excretion to reducing the toxicity of absorbed DHP (6).

Both isomers of DHP (3,4 and 2,3) are regarded as goitrogenic (60). However, DHP is not only excreted in the free form but also as a glucuronide conjugate in urine (26, 31). Free 3,4-DHP and its glucuronide conjugate are both goitrogenic, but the conjugate appears less toxic when administered intravenously, although relatively little work has been done on its bioactivity (29). It is likely that conjugated DHP has a lower affinity for thyroid tissue and is cleared more rapidly by the kidneys (29). Renal excretion of 2,3-DHP also appears to be enhanced when the molecule is chelated with metal ions (61). It was thought that approximately one-third of DHP excreted in the urine was in a conjugated form, but recent research has shown that the majority of excreted 2,3-DHP is mainly in a conjugated form, whereas 3,4-DHP is primarily excreted as the unconjugated form at low or negligible levels (6, 62). Historically, the levels of DHP excretion in urine have been underestimated until recently when it was found that much of the conjugated DHP was not accounted for unless the urine was rigorously acid-hydrolyzed (63). These observations differ from the earlier work that indicated DHP excretion in urine declined rapidly after inoculation with *S. jonesii*, and 2,3 DHP was a transient intermediate in the complete degradation of 3,4 DHP to non-toxic products (38, 58). Conversion of 3,4-DHP to 2,3-DHP occurs during the first week of exposure to leucaena, and 2,3-DHP becomes the dominant isomer in urine, but the rate of this microbial transformation increases over several weeks (56, 62). Therefore, the main detoxification process for mimosine involves both microbial degradation in the gut and conjugation of the 2,3-DHP isomer in the liver.

## CURRENT STATUS

The notion that *S. jonesii* was absent on the Australian continent prior to the introduction of a rumen inoculum from a Hawaiian goat in the 1980s seems unlikely. The early reports of rapid and apparent complete degradation of DHP following inoculation with *S. jone*sii have not been replicated in recent studies, even though *S. jonesii* is present in the rumen. Early studies relied on the semi-quantitative colorimetric detection of DHP metabolites in urine or *in vitro* mixed cultures as evidence for the presence of *S. jonesii* (37), whereas quantitative HPLC analysis and an improved acid hydrolysis extraction of urine, along with quantification of the unhydrolyzed conjugated DHP used recently, is more definitive and demonstrates the recovery of metabolites in urine was previously underestimated (6, 62). Since the first isolation of *S. jonesii*, the data set of Synergistota 16S rRNA gene sequences and genomes has expanded, and highly sensitive PCR-based detection assays have been developed, which helped confirm the widespread presence of *S. jonesii* in herbivore gastrointestinal (GI) tracts in many countries, including Australia (47). These molecular techniques have also demonstrated that there are genetic variants of the type strain 78–1 in all countries tested. Unfortunately, strain 78–1 remains the only genetic variant of *S. jonesii* in culture, so the potential for phenotypic variation between strains has not been tested but remains a distinct possibility. Early reports of toxicity in Australian ruminants prior to inoculation may be due to less potent indigenous strains that required adaptation to the toxin not previously in the environment. The few studies that have been reported show that the metabolism of the 3,4- and 2,3-DHP isomers is regulated by their concentration, and activity can be lost due to the lack of the substrate. Future research should endeavor to isolate new strains of *S. jonesii*, so comparative studies of DHP degradation, genomics, and transcriptomics can be performed, which might explain some of the apparent toxicity that has been observed in different countries.

## FLUOROACETATE TOXICITY

**Fig 7:**
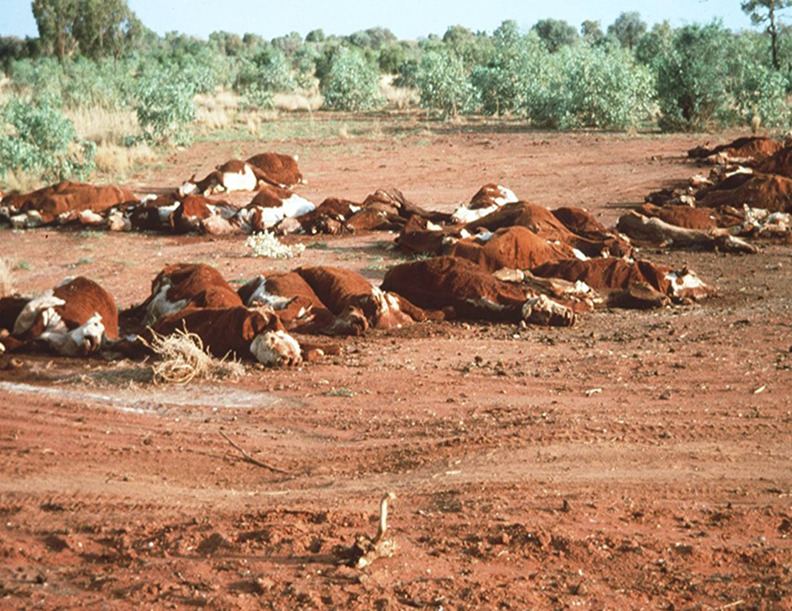
Acute poisoning and death of cattle in northern Australia by fluoroacetate-producing plants (*Acacia georginae* in background).

Plants that contain FA grow worldwide and cause losses in productivity and acute death in ruminant livestock (Fig. 7). The southern continents of tropical and sub-tropical Australia, Africa, and South America are the main geographical regions where these plants grow. In Australia, the main plants responsible for FA toxicity are *Acacia georginae* and *Gastrolobium* species (64). Fluoroacetate causes acute toxicity by interfering with the tricarboxylic acid cycle, which is key to ATP energy generation in the mitochondria of higher organisms. FA forms fluoroacetyl CoA, which is converted to fluorocitrate that strongly binds to the aconitase enzyme, causing citrate accumulation and disturbed cellular respiration (65, 66).

### Anaerobic microbial detoxification

A novel approach to solving the problem of FA toxicity in Australian ruminants was commenced during the 1990s, which sought to construct genetically modified (GM) rumen bacteria capable of degrading FA. This approach was justified on the basis that anaerobic metabolism of FA did not occur in rumen microorganisms. The GM rumen bacteria were constructed using strains of the rumen bacterium *Butyrivibrio fibrisolvens*, which were transformed with the plasmid pBHf carrying the fluoroacetate dehalogenase gene (DelH1) from the aerobic soil organism *Delftia acidovorans* strain B (67). Two *in vivo* studies conducted in sheep and cattle, respectively, inoculated with these recombinant bacteria showed a significant reduction in toxicity (68, 69). Although this approach showed significant potential, it was not adopted in Australia as the approvals for the release of genetically modified organisms were not sought or granted due to strict government regulations. However, on reflection, it was noted that publications on the ability of the rumen to degrade fluoroacetate did not exist. In response, a renewed effort was mounted to examine whether rumen microorganisms that could metabolize FA already existed in nature (11, 12). Consequently, two bacteria have been isolated from an Australian bovine rumen using enrichment cultures containing fluoroacetate as the main carbon source, followed by selection on anaerobic agar plates containing the toxin, but other uncultured bacteria may exist (11, 12). These bacteria *Cloacibacillus porcorum* strain MFA1 and *Pyramidobacter piscolens* strain C12-8, which are available from the CSIRO culture collection, both belong to the Synergistota phylum (Fig. 4). Strain MFA1 is rod-shaped, with a true gram-negative wall and electron-dense granules distributed throughout the cell (Fig. 3). Strain C12-8 cells vary between coccoid and coccobacillus in shape, having an unusual cell envelope, with only one membrane and no obvious external wall (Fig. 3), which is not characteristic of either gram-negative or gram-positive cells. Using PCR detection, gut samples from a range of herbivores in Australia showed the wide distribution of strain MFA1 and closely related strains in the gut of cattle, kangaroos, wallabies, and emus but in relatively low numbers (11). A more sensitive and specific PCR assay was employed in a later survey of northern Australia cattle properties, which indicated that strain C12-8 and other FA-degrading bacteria affiliated with *C. porcorum* strain MFA1 were endemic to cattle in this part of Australia. Quantitative PCR showed C12-8 was present in the range of 10^4^–10^6^ cells/mL rumen fluid, while strain MFA1 appeared to be at lower numbers (12).

Both *C. porcorum* strain MFA1 and *P. piscolens* strain C12-8 are able to produce fluoride and acetate as the end products of fluoroacetate metabolism (11, 12, 64). Growth of both these bacteria was stimulated by protein hydrolysates, with a preference for the use of hydrophilic amino acids. Energy appears to be generated in Synergistota from the metabolism of these amino acids (70). The use of amino acids is enhanced, and hydrogen as well as formate are consumed when fluoroacetate is co-metabolized, leading to the conclusion that FA is degraded by the process of reductive dehalogenation (dehalorespiration and organohalide respiration) (11). Other Synergistota bacteria also showed similar enhanced amino acid metabolism when grown in the presence of H_2_ scavengers such as methanogens (71–73).

**Fig 8:**
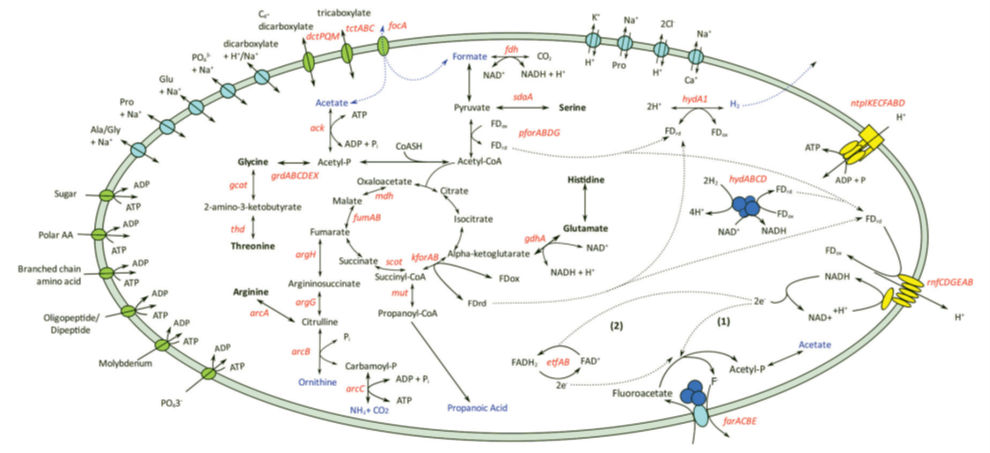
Energy conservation pathways and solute transport of *C. porcorum* strain MFA1. Amino acids utilized by strain MFA1 were shown in bold, and metabolite products were highlighted in blue. Genes identified from the genome were labeled in red. Genes annotated within amino acid metabolic pathways are argininosuccinate lyase (*argH*), argininosuccinate synthetase (*argG*), arginine deiminase (*arcA*), ornithine carbamoyltransferase (*arcB*), carbamate kinase (*arcC*), serine ammonia-lyase (*sdaA*), L-threonine 3-dehydrogenase (*thd*), glycine C-acetyltransferase (*gcat*), glycine reductase (*grdABCDEX*), and glutamate dehydrogenase (*gdhA*). Genes involved with energy or metabolic product formations are formate dehydrogenase (*fdh*), pyruvate:ferredoxin oxidoreductase (*pforABC*), succinyl-CoA:oxoacid transferase (*scot*), malate dehydrogenase (*mdh*), fumarase (*fumAB*), α-ketoglutarate:ferredoxin oxidoreductase (*kforAB*), methylmalonyl-CoA mutase (*mut*), acetate kinase (*ack*), electron-transferring flavoprotein (*etfAB*), iron-only hydrogenase (*hydA1*), NADH-dependent FeFe-hydrogenase (*hydABC*), “*Rhodobacter*-nitrogen fixation” (*rnfCDGEAB*), and archaeal/vacuolar-type H+^+^-ATPase (*ntpABCDEFGHI*). Enzymatic reactions with secondary active transporters are shown in light blue, while other transporters, including ATP-binding cassette (ABC) transporter, tripartite ATP-independent periplasmic (*dctPQM*), tripartite tricarboxylate transporter (*tctABC*), and formate transporter, are shown in green. ABC transporters are indicated by the exchange of ATP with ADP. (Reproduced from reference 19, published under the terms of the Creative Commons License**,** https://creativecommons.org/licenses/by/4.0/.)

The molecular basis of reductive dehalorespiration (organohalide respiration) is not well understood and has been studied mainly in bacteria whichthat use chlorinated compounds as terminal electron acceptors for dehalogenation (74). However, a common feature of the process is that membrane-bound reductive dehalogenases (RDases), in concert with other components, facilitate the release of halide ions, thus generating cellular energy. The mechanism for FA degradation in the Synergistota bacteria *C. porcorum*, *Cloacibacillus evryensis*, and *P. piscolens* was investigated using molecular genomics to identify the basis for the metabolism of FA. Comparative genome and transcriptomic analyses identified a candidate operon (*farACEB*, fluoroacetate reductase) in all three bacteria, which consists of four genes encoding a secondary active transporter that transports FA, two substrate-binding components of the glycine reductase substrate-specific protein complex B, and an iron-sulphfur oxidoreductase that activates the GrdB-like protein (19). Complete amino acid fermentation pathways detected in the *C. porcorum* strain MFA1 genome from which energy is generated are shown in Fig. 8.

*C. porcorum*, *Cloacibacillus evryensis*, and *P. piscolens* were investigated using molecular genomics to identify the basis for the metabolism of FA. Comparative genome and transcriptomic analysis identified a candidate operon (*farACEB,* fluoroacetate reductase) in all three bacteria which consists of four genes encoding a secondary active transporter that transports FA, two substrate binding components of the glycine reductase (GR) substrate-specific protein complex B, and an iron-sulphur oxidoreductase that activates the GrdB-like protein (19). Complete amino acid fermentation pathways detected in the *C. porcorum* strain MFA1 genome from which energy is generated are shown in Fig. 8.

This novel mechanism for MFA metabolism involving glycine reductase enzymes suggests that there is substrate plasticity in amino acid-reducing enzymes, which includes xenobiotic fluoroacetate.

## CONCLUSION

These studies of rumen bacteria, which metabolize the plant secondary compounds DHP and FA, lend further support to the notion that Synergistota bacteria have differing abilities to use xenobiotics as electron acceptors to facilitate fermentation of amino acids, which are their primary carbon source. All members of the phylum display a similar metabolic niche of amino acid fermentation, but their ability to use a range of substrates as electron acceptors that are difficult for other bacteria to degrade anaerobically allows them to exploit and compete in varied physical niches.

Strategies that reliably reduce toxicity from DHP and fluoroacetate in ruminants through the ability of Synergistota bacteria to degrade these compounds are still dependent upon a thorough knowledge of the physiological requirements of these bacteria and the molecular regulation of these metabolic pathways. This will ensure that the populations of these bacteria can be boosted to the highest levels that are ecologically possible while providing substrates that optimize the regulation of genes involved in the efficient degradation of the toxins.

## EDITOR'S NOTE

**Ed. Note:** A potential conflict of interest was identified during review of this paper, and the Editor in Chief performed an additional final review of the paper.
